# Unsafe abortion and associated factors among reproductive aged women in Sub-Saharan Africa: a protocol for a systematic review and meta-analysis

**DOI:** 10.1186/s13643-018-0775-9

**Published:** 2018-08-25

**Authors:** Merhawi Gebremedhin, Agumasie Semahegn, Tofik Usmael, Gezahegn Tesfaye

**Affiliations:** 1 College of Health and Medical Sciences, Haramaya University, PO Box 235, Harar, Ethiopia; 2 IPAS Ethiopia, Addis Ababa, Ethiopia; 3 School of Public Health, College of Health Science, University of Ghana, Legon, Accra, Ghana

**Keywords:** Systematic review and meta-analysis, Unsafe abortion, Sub-Saharan Africa

## Abstract

**Background:**

Unsafe abortion is a neglected public health problem contributing for 13% of maternal death worldwide. In Africa, 99% of abortions are unsafe resulting in one maternal death per 150 cases. The prevalence of unsafe abortion is associated with restricted abortion law, poor quality of health service, and low community awareness. Hence, the aim of this systematic review and meta-analysis is to identify and summarize the available evidence to generate an abridged evidence on the prevalence of unsafe abortion and its associated factors in Sub-Saharan Africa.

**Methods:**

The development of the systematic review methodology has followed the procedural guideline depicted in the preferred reporting items for systematic review and meta-analysis protocol statement. Observational studies that have been conducted from January 1, 1994, up to December 31, 2017, in Sub-Saharan African countries will be included in the systematic review and meta-analysis. MEDLINE (via PubMed), EMBASE, CINAHL, and PopLine will be searched to retrieve available studies. Relevant studies will be retrieved using the search strings applied to different sources. The Joanna Briggs Institute quality assessment tool will be used to critically appraise the methodological robustness and validity of the finding to avoid erroneous data due to confounded or biased statistics. Data extraction template will be prepared to record abstracted information from selected studies. The selection of relevant studies, data extraction, and quality assessment of studies will be carried out by two authors. Meta-analysis using Mantel–Haenszel random effects model will be carried out. The presence of heterogeneity between studies will be checked using the *I*^2^ value.

**Discussion:**

Unsafe abortion is not yet reduced significantly in Sub-Saharan Africa, and maternal death rate due to unsafe abortion remains high. Currently, there is a gap in availability of abridged evidence on unsafe abortion and this negatively influenced the current service delivery. This finding will help stakeholders to design appropriate strategy. The finding of this systematic review and meta-analysis will be helpful to inform policy-makers, programmers, planners, clinician’s decision making, researchers, and women clients at large.

**Systematic review registration:**

PROSPERO 2017: CRD42017081437.

**Electronic supplementary material:**

The online version of this article (10.1186/s13643-018-0775-9) contains supplementary material, which is available to authorized users.

## Background

Unsafe abortion is entirely preventable. However, it remains pandemic and serious public health issue worldwide [1–4]. The World Health Organization (WHO) defines unsafe abortion as a procedure of pregnancy termination either by persons lacking the necessary skills or in an environment that does not conform to minimal medical standards or both [5]. Unsafe abortion is a neglected problem of health care in developing countries [4]. Despite technological advancements in health care, unsafe abortion remained essentially unchanged worldwide [6]. Unsafe abortion is identified as one of the major cause of maternal morbidity and mortality [7]. In Sub-Saharan Africa (SSA), abortion is more common and it tends to be clandestine and unsafe that has a substantially contribution to maternal mortality [8].

Worldwide, 210 million women become pregnant each year. Of these, 80 million pregnancies are unplanned. Out of these, 46 million pregnancies terminated each year, and 19 million ends with unsafe abortion [1–4]. More than 97% of unsafe abortions take place in developing countries [2, 4, 9, 10]. Globally, unsafe abortion increased from 44% in 1995 to 49% in 2008 [2, 10]. In 2000, the WHO estimates that one in ten pregnancies end up with unsafe abortion, giving one unsafe abortion to seven live births ratio. Likewise, 68,000 women die due to unsafe abortion each year, and the risk of maternal death is high in developing countries (1 in 270 unsafe abortion) [4].

The maternal death associated with unsafe abortion was 37 deaths per 100,000 live births in SSA, 23 per 100,000 in Latin America and the Caribbean, and 12 per 100,000 in South Asia [1]. The WHO (2008) estimates that unsafe abortion contributes for 13% of maternal death, worldwide. However, in Africa, the contribution of unsafe abortion is too high which is 2.4 million unsafe abortions occurred in eastern Africa in 2008. Globally, 40% of reproductive aged women live in countries with highly restrictive abortion law [11]. In Africa, over 4 million unsafe abortions are carried out yearly; mostly on poor, rural, and young women lacking information on availability of safe abortion care. About 99% of all abortions carried out in Africa are unsafe, and the risk of maternal death from an unsafe abortion is one in every 150 procedures which is the highest in the world [12, 13].

The prevalence of unsafe abortion is attributable to poverty, social inequity, and denial of women’s human rights [1]. Countries with restricted abortion or where abortions are clandestine and unsafe, its consequences to women’s health are harmful, particularly for young, poor, and low-education women [3, 14]. Unsafe abortion is practiced using different methods such as use of oral and injectable items, vaginal preparations, intrauterine foreign bodies, and trauma to the abdomen [13]. Significant proportion of women (20–50%) with unsafe abortion develop complications that lead to hospital admission. These complications include hemorrhage, sepsis, peritonitis, and trauma to the cervix, vagina, uterus, and abdominal organs [2]. The Sustainable Development Goals (SDG) aim to reduce global maternal mortality ratio from 216 to 70 per 100, 000 live births by 2030. Therefore, in order to contribute to this goal, developing countries need to legalize abortion and improve health care system to reduce abortion-related maternal deaths [2, 15]. Hence, the main purpose of this systematic review and meta-analysis is to identify and summarize the available evidence to determine prevalence of unsafe abortion among women in the reproductive age and associated factors in SSA.

## Methods

### Development review protocol and registration

The development of the review methodology has followed the procedural guideline that was endorsed by the preferred reporting items for systematic review and meta-analysis protocol (PRISMA-P) statement [16], and all of the items in the checklist were completed (see Additional file 1). The review protocol has been registered in international prospective register of systematic reviews (PROSPERO) with trial registration number (CRD42017081437).

### Data source and searching strategies

The search of studies will be carried out by (MG and GT). Published and unpublished studies written in English will be retrieved and included into the review process. Databases such as MEDLINE (via PubMed), EMBASE, CINAHL, and POPLINE will be searched for studies that had been conducted since January 1, 1994. Relevant sources such as Google search engine, Google scholar, and WHO websites will be searched. In addition, experts on the field will be consulted to retrieve unpublished studies. The year 1994 was chosen because the international community recognized the pressing need to address unsafe abortion at the International Conference on Population and Development (ICPD) in the year 1994 [17] and many African countries endorsed semi-restricted abortion law since 1994 [18]. The search strings will emerge from the following keywords (unsafe abortion, induced abortion, abortion, Sub-Saharan Africa, or African South of Sahara). Depending on the specific requirement of the database, the search string will be modified, and relevant studies using search strings will be identified. The combinations of free keywords and MeSH (medical sub-headings) will be extensively used in the search process. The reference lists of relevant studies will also be reviewed for sources that may have been missed in the database search. The search strategy developed for selected database is attached (see Additional file 2).

### Eligibility criteria

All observational studies (cross-sectional, case-control, and cohort) and survey reports will be included in the systematic review. However, case reports, case series, commentaries, and editorials will be excluded from the review. All studies with primary objective to determine the prevalence of unsafe abortion and/or its associated factors among reproductive aged women in Sub-Saharan Africa will be considered [8].

We will consider studies that defined unsafe abortion based on WHO definition [19]; WHO defines unsafe abortion as a procedure of pregnancy termination either by persons lacking the necessary skills or in an environment that does not conform to minimal medical standards or both. We will also include community or facility-based studies that used either primary or secondary data. We will include studies that had mainly reported prevalence of unsafe abortion and its associated factors. However, as far as our primary aim is to determine the prevalence of unsafe abortion, studies that reported only prevalence of unsafe abortion but not associated factors will be included. In addition, studies that at least had test statistics that measured association between predictor variables with unsafe abortion will be considered to identify the associated factors. The studies should have a crosstab showing difference in prevalence of unsafe abortion in the categories of the exposure variables. We will exclude studies that only investigated unsafe abortion with qualitative approach. If we come across studies that have both quantitative and qualitative study finding, we will only consider the quantitative findings.

### Selection of studies

We will export all retrieved studies into the Endnote citation management software [20]. Initially, duplicated studies will be removed from the citation manger. The two authors (MG and AS) will independently screen the studies based on information contained in the titles and abstract based on the inclusion criteria. Studies that clearly mentioned unsafe abortion among reproductive aged women will be selected for the next step of evaluation. Consequently, studies that have been eligible based on their title and abstract will be further screened by GT and TU. Based on title and abstract assessment, the studies will be classified as included, excluded, and undecided studies. For studies that will be categorized as included and undecided, we will further examine and evaluate full texts of the studies for eligibility. The full-text screening will be carried out by GT and AS. Studies that will not be eligible based on the full-text assessment will be excluded and reasons will be described for their exclusion. Studies that will pass through this selection process will be included in qualitative and quantitative synthesis. During screening of the studies, any disagreement among reviewers will be resolved by discussion and reach common understanding. The study selection process flow diagram is adapted from PRISMA guideline [16] (see Additional file 3).

### Quality assessment

Studies will be critically evaluated for their validity of the findings. To determine the methodological robustness and validity of the findings of the studies, we will use the JBI (Joanna Briggs Institute) tool for assessing the quality of evidence. Particular attention will be given to clear statement of the objective of the study, sampling techniques, precision of measurement of outcomes of interest and exposure variables, as well as documentation of sources of bias or confounding. The two review authors (GT and AS) will check the scientific quality of the studies independently using quality assessment tool mentioned above. In case of uncertainties, it will be resolved by joint discussion between them.

### Data extraction

Data extraction template will be constructed on Microsoft Excel (2013). The two authors (MG and TU) will extract data systematically and stored using data extraction form. Piloting of the data extraction form will be carried out before the beginning of the actual data extraction. Study description tables will be used to record the type of study design, aim, sample size, primary outcomes of interest (prevalence of unsafe abortion), and secondary outcome (associated factors). Numerical data (frequency) will also be extracted and recorded in Microsoft Excel sheet. The systematic review and meta-analysis working group will contact authors of the studies to request for details through email in case of missing data, incomplete report, or any uncertainties.

### Data synthesis and statistical analysis

The data will first be presented using narrative synthesis of the included studies. A summary table will be prepared to describe characteristics (author-date, country, design, aim, sampling method, sample size, response rate, and key findings) of the included studies. The presence of statistical heterogeneity will be checked by using the Cochran Q test. The level of heterogeneity among the studies will be quantified using the *I*^2^ statistics where substantial heterogeneity will be assumed if the *I*^2^ value is ≥ 60%. We will also check the presence of publication bias using funnel plot if more than ten studies are included. We will also do Egger’s and Beggar’s test to check publication bias [21]. To pool prevalence of unsafe abortion, we will conduct meta-analysis using Comprehensive Meta-analysis software [22]. We will use the random effects model and the raw numerical data (number of unsafe abortions (*n*) and total sample size (*N*)) from each study. We hypothesize that the legal and illegal status of abortion influences the magnitude of unsafe abortion. Therefore, we will conduct sub-group analysis of the prevalence of unsafe abortion based on countries abortion legal status. Moreover, we will use adjusted, and if none available unadjusted, odds ratios to assess the association between risk factors and unsafe abortion.

## Discussion

The aim of this systematic review and meta-analysis is to synthesis research findings on the prevalence of unsafe abortion and its associated factor in SSA. Even though evidence [23] indicates that unsafe abortion is not showing reduction in SSA, there is no systematically reviewed evidence that show the overall prevalence of unsafe abortion and influencing factors in the region. Moreover, currently, there is a gap in the availability of complete data on unsafe abortion and this can negatively influence the prevailing service delivery [24]. Establishing reliable evidence on the magnitude of unsafe abortion are generally challenging especially in countries where access to abortion is legally restricted. Whether legal or illegal, induced abortion is usually stigmatized and frequently censured by political, religious, or other cultural issues. Hence, under-reporting is routine even in countries where abortion is legally available [25].

The magnitude of unsafe abortion can be measured using different approaches namely absolute numbers, incidence ratio, incidence rate, mortality ratio, and case fatality rate. However, absolute number of unsafe abortions cannot be used to compare the magnitude in different regions or sub-regions because of difference in population size. In our analysis, ratios and rates will be used to allow inter or intra comparisons of nation(s) [4]. Worldwide report indicates that the rate of unsafe abortion is not decreased at the same pace with that of safe abortion. Unsafe abortions changed very little: from 19.9 million in 1995 to 19.7 million in 2003 [26]. But there is no specific data that indicates the prevalence of unsafe abortion to support the current initiative to reduce the rate of unsafe abortion in the region.

Evidence indicates that maternal mortality ratio secondary to unsafe abortion is 950 times higher in SSA (520) than in the USA (0.6) per 100,000 live births, respectively. The burdens of unsafe abortion and its associated maternal mortality are disproportionately higher for women in Africa than in any other developing region [27]. Its share of global unsafe abortions was 29%, and more seriously, 62% of all deaths related to unsafe abortion occurred in Africa in 2008 [28]. In places where laws and policies allow abortion under broad indications, the incidence of and mortality from unsafe abortion are reduced to a minimum [28].

Meanwhile, unsafe abortion affects the health of millions of women predominantly the poor, illiterate, and those living in rural areas, and hence, knowing the prevailing situation of unsafe abortion could help develop appropriate programs that potentially circumvent its occurrence. Experts proposed that expanding effective modern contraceptive methods, making abortion legal with accessible safe abortion services, and improving the quality of post abortion care would reduce the magnitude of unsafe abortion, its associated maternal mortality and morbidity, and cost of post abortion services [26, 29]. Systematic review conducted in SSA showed that care givers in general were uncertain about the legal status of abortion in their countries, with majority of them having negative feeling towards induced abortion and only some of the health care providers perceived the legalization of abortion as a positive step [1].

Subsequently, it remains important to assess the magnitude of unsafe abortion and its associated factors in SSA so as to inform the development of appropriate programs and policy that would have an impact in reducing maternal morbidity and mortality in the region. The finding from this systematic review will be important for national governments and nongovernmental organizations in the health sector of the individual countries of the region to give emphasis on the main factors that drive unsafe abortion. Moreover, this finding will also help governments and other health development partners to expand and improve family planning services, to further advocate for legalization of abortion and increase accessibility and availability of abortion services in order to improve women’s health and well-being [4]. Therefore, the finding of this systematic review and meta-analysis will be used to inform policy-makers, health programmers, clinicians’ decision making, researchers, human right activist, and women clients at large.

## Additional files

Additional file 1: PRISMA-P checklist. (DOC 84 kb)

Additional file 2: Sample search strategy using search strings. (PDF 19 kb)

Additional file 3: Diagramatic presentation of the studies selection process for systematic review. (DOCX 36 kb)

## Abbreviations

ICPD: International Conference on Population and Development
JBI: Joanna Briggs Institute
PRISMA: Preferred reporting items for systematic review and meta-analysis
SDG: Sustainable Development Goals
SSA: Sub-Saharan Africa
WHO: World Health Organization
