# Supplementary material for: Unsafe abortion and associated factors among reproductive aged women in Sub-Saharan Africa: a protocol for a systematic review and meta-analysis
Source: Syst Rev. 2018 Aug 25;7:130. doi: 10.1186/s13643-018-0775-9 (PMC6109307; doi:10.1186/s13643-018-0775-9)
Supplement: Supplementary file 2 — Sample search strategy using search strings. (PDF 19 kb) [file 13643_2018_775_MOESM2_ESM.pdf]

Search Strategy

Title of the review: Unsafe abortion and associated factors among reproductive aged women in sub-Saharan Africa: A protocol for a systematic review and meta-analysis
Date of final search: April 7, 2018

**Data base**	**Search filters (Year=01/01/1994 to 31/12/2017, Language=English), DATE OF THIS SEARCH [April 7, 2018]**	
	**Combined search phrase**	**Result**
**PubMed**	((((((unsafe abortion[All Fields] OR unsafe abortions[All Fields]) OR (induced abortion[All Fields] OR induced abortions[All Fields])) OR (legal abortion[All Fields] OR legal abortions[All Fields])) OR ("abortion, spontaneous"[MeSH Terms] OR ("abortion"[All Fields] AND "spontaneous"[All Fields]) OR "spontaneous abortion"[All Fields] OR "miscarriage"[All Fields])) OR (abortion[All Fields] OR abortion'[All Fields] OR abortion"[All Fields] OR abortion's[All Fields] OR abortion1[All Fields] OR abortional[All Fields] OR abortionist[All Fields] OR abortionist's[All Fields] OR abortionists[All Fields] OR abortions[All Fields] OR abortions'[All Fields] OR abortionsthese[All Fields])) AND (((Magnitude[All Fields] OR ("epidemiology"[Subheading] OR "epidemiology"[All Fields] OR "prevalence"[All Fields] OR "prevalence"[MeSH Terms])) OR ("epidemiology"[Subheading] OR "epidemiology"[All Fields] OR "incidence"[All Fields] OR "incidence"[MeSH Terms])) OR ("trends"[Subheading] OR "trends"[All Fields]))) AND (Sub-Saharan[All Fields] OR ("africa south of the sahara"[MeSH Terms] OR ("africa"[All Fields] AND "south"[All Fields] AND "sahara"[All Fields]) OR "africa south of the sahara"[All Fields] OR ("sub"[All Fields] AND "saharan"[All Fields] AND "africa"[All Fields]) OR "sub saharan africa"[All Fields]) OR (("poverty"[MeSH Terms] OR "poverty"[All Fields] OR ("low"[All Fields] AND "income"[All Fields]) OR "low income"[All Fields]) AND country[All Fields]) OR ("developing countries"[MeSH Terms] OR ("developing"[All Fields] AND "countries"[All Fields]) OR "developing countries"[All Fields] OR ("developing"[All Fields] AND "country"[All Fields]) OR "developing country"[All Fields]) OR ("angola"[MeSH Terms] OR "angola"[All Fields]) OR ("benin"[MeSH Terms] OR "benin"[All Fields]) OR ("botswana"[MeSH Terms] OR "botswana"[All Fields]) OR ("burkina faso"[MeSH Terms] OR ("burkina"[All Fields] AND "faso"[All Fields]) OR "burkina faso"[All Fields]) OR ("burundi"[MeSH Terms] OR "burundi"[All Fields]) OR ("cameroon"[MeSH Terms] OR "cameroon"[All Fields]) OR ("cabo verde"[MeSH Terms] OR ("cabo"[All Fields] AND "verde"[All Fields]) OR "cabo verde"[All Fields] OR ("cape"[All Fields] AND "verde"[All Fields]) OR "cape verde"[All Fields]) OR ("central african republic"[MeSH Terms] OR ("central"[All Fields] AND "african"[All Fields] AND "republic"[All Fields]) OR "central african republic"[All Fields]) OR ("chad"[MeSH Terms] OR "chad"[All Fields]) OR ("comoros"[MeSH Terms] OR "comoros"[All Fields]) OR ("congo"[MeSH Terms] OR "congo"[All Fields]) OR ("cote d'ivoire"[MeSH Terms] OR ("cote"[All Fields] AND "d'ivoire"[All Fields]) OR "cote d'ivoire"[All Fields]) OR ("djibouti"[MeSH Terms] OR "djibouti"[All Fields]) OR ("equatorial guinea"[MeSH Terms] OR ("equatorial"[All Fields] AND "guinea"[All Fields]) OR "equatorial guinea"[All Fields]) OR ("eritrea"[MeSH Terms] OR "eritrea"[All Fields]) OR ("ethiopia"[MeSH Terms] OR "ethiopia"[All Fields]) OR ("gabon"[MeSH Terms] OR "gabon"[All Fields]) OR ("gambia"[MeSH Terms] OR "gambia"[All Fields]) OR ("ghana"[MeSH Terms] OR "ghana"[All Fields]) OR ("guinea"[MeSH Terms] OR "guinea"[All Fields]) OR ("guinea-bissau"[MeSH Terms] OR "guinea-bissau"[All Fields] OR ("guinea"[All Fields] AND "bissau"[All Fields]) OR "guinea bissau"[All Fields]) OR ("kenya"[MeSH	**381**

	Terms] OR "kenya"[All Fields]) OR ("lesotho"[MeSH Terms] OR "lesotho"[All Fields]) OR ("liberia"[MeSH Terms] OR "liberia"[All Fields]) OR ("madagascar"[MeSH Terms] OR "madagascar"[All Fields]) OR ("malawi"[MeSH Terms] OR "malawi"[All Fields]) OR ("mali"[MeSH Terms] OR "mali"[All Fields]) OR ("mauritania"[MeSH Terms] OR "mauritania"[All Fields]) OR ("mauritius"[MeSH Terms] OR "mauritius"[All Fields]) OR ("mozambique"[MeSH Terms] OR "mozambique"[All Fields]) OR ("namibia"[MeSH Terms] OR "namibia"[All Fields]) OR ("niger"[MeSH Terms] OR "niger"[All Fields]) OR ("nigeria"[MeSH Terms] OR "nigeria"[All Fields]) OR ("reunion"[MeSH Terms] OR "reunion"[All Fields]) OR ("rwanda"[MeSH Terms] OR "rwanda"[All Fields]) OR (Sao[All Fields] AND Tome[All Fields]) AND Principe[All Fields] OR ("senegal"[MeSH Terms] OR "senegal"[All Fields]) OR ("seychelles"[MeSH Terms] OR "seychelles"[All Fields]) OR ("sierra leone"[MeSH Terms] OR ("sierra"[All Fields] AND "leone"[All Fields]) OR "sierra leone"[All Fields]) OR ("somalia"[MeSH Terms] OR "somalia"[All Fields]) OR ("south africa"[MeSH Terms] OR ("south"[All Fields] AND "africa"[All Fields]) OR "south africa"[All Fields]) OR ("sudan"[MeSH Terms] OR "sudan"[All Fields]) OR ("swaziland"[MeSH Terms] OR "swaziland"[All Fields]) OR ("tanzania"[MeSH Terms] OR "tanzania"[All Fields]) OR ("togo"[MeSH Terms] OR "togo"[All Fields]) OR ("uganda"[MeSH Terms] OR "uganda"[All Fields]) OR (Western[All Fields] AND ("africa, northern"[MeSH Terms] OR ("africa"[All Fields] AND "northern"[All Fields]) OR "northern africa"[All Fields] OR "sahara"[All Fields])) OR ("zambia"[MeSH Terms] OR "zambia"[All Fields]) OR ("zimbabwe"[MeSH Terms] OR "zimbabwe"[All Fields])) AND (("1994/01/01"[PDAT] : "2017/12/31"[PDAT]) AND "humans"[MeSH Terms] AND English[lang])	
