# Supplementary material for: Unsafe abortion and associated factors among reproductive aged women in Sub-Saharan Africa: a protocol for a systematic review and meta-analysis
Source: Syst Rev. 2018 Aug 25;7:130. doi: 10.1186/s13643-018-0775-9 (PMC6109307; doi:10.1186/s13643-018-0775-9)
Supplement: Supplementary file 3 — Diagramatic presentation of the studies selection process for systematic review. (DOCX 36 kb) [file 13643_2018_775_MOESM3_ESM.docx]

| Studies collected using other relevant sources (email request, thesis and USB) (n)  Potentially relevant studies obtain through database search (n)  Duplicated Studies removed (n)  Number of studies after removal of duplications (n)  Papers excluded based on reviewing the title and abstract sections (n)  Studies included based on Title and abstract (n)  Studies eligible for the whole body review for eligibility to be included to the final review (n)  Exclude from the review after assessing the full text of the paper. The papers did not clearly reported prevalence and associated factors (n)  Studies included in the systematic review (n)  Studies included for meta-analysis (n) |
| --- |
